# Removal of Pb(II) ions by cellulose modified-LaFeO_3_ sorbents from different biomasses

**DOI:** 10.1186/s13065-023-01066-2

**Published:** 2023-11-04

**Authors:** Shimaa M. Ali, Mohamed A. El Mansop, Ahmed Galal, Soha M. Abd El Wahab, Wafaa M. T. El-Etr, Hanaa A. Zein El-Abdeen

**Affiliations:** 1 Chemistry Department, Faculty of Science, Cairo University, Giza, 12613 Egypt; 2 Physics Department, Faculty of Science, Cairo University, Giza, 12613 Egypt; 3 Soil, Water and Environmental Research Institute, Agriculture Research Center (ARC), Giza, 12613 Egypt

**Keywords:** LaFeO_3_ perovskite, Cellulose-modified synthesis method, Biomass type, Adsorption, Water decontamination

## Abstract

LaFeO_3_ perovskite is prepared by the cellulose-modified microwave-assisted citrate method using two different biomasses as a cellulose source; rice straw (RS) and banana peel (BP). The prepared samples are assigned as LaFeO_3_/cellulose-RS and as LaFeO_3_/cellulose-BP, respectively. Raman Spectra prove the presence of perovskite and cellulose phases, as well as biochar resulted from the thermal treatment of the cellulose. LaFeO_3_/cellulose-RS has a cauliflower morphology while, two phases are observed for LaFeO_3_/cellulose-BP, mesoporous cellulose phase and octahedral LaFeO_3_ nanoparticles as shown by scanning electron microscope (SEM) images. LaFeO_3_/cellulose-BP has higher porosity and larger BET surface area than LaFeO_3_/cellulose-RS. Both samples are applied for the removal of Pb(II) ions from aqueous solution by adsorption. The adsorption follows Langmuir isotherm, with maximum adsorption capacities of 524 and 730 mg/g for LaFeO_3_/cellulose-RS and LaFeO_3_/cellulose-BP, respectively. Cellulose precursors from different biomasses affect structural and morphological properties of LaFeO_3_/cellulose samples as well as the sorption performance for Pb(II) ions. BP is more recommended than RS, as a biomass, in the present study.

## Introduction

A considerable amount of toxic Pb(II) ion is released to the environment and pollutes water as an industrial effluent of many processes such as battery and chemicals manufacture, refining, and automobile maintenance [1]. Pb(II) ion is toxic and has the most global abundance among heavy metal ions [2]. It accumulates in the human body, causing dangerous diseases such as, kidneys and brain damage, anemia, cancer, and many others [3]. The removal of Pb(II) ions, and other toxic metal ions, from wastewater is an essential and critical issue [4, 5]. There are several methods that can be applied for example, adsorption [6], coagulation [7], membrane filtration [8], chemical precipitation [9], electrodialysis [10], etc. The method used in this work is adsorption, not only because it is simple and cost-effective method but also, due to the progress in materials science, which permits researchers to prepare novel and efficient sorbents materials. An economically trend, that has been recently and extensively applied, is the use of waste for waste, i.e. the use of agricultural waste or biomass to prepare sorbents for the removal of toxic pollutants from wastewater. Due to simplicity, availability, and low cost, raw and modified biomasses are highly recommended as efficient sorbents, for example, Banana and orange peels, rice straw, potato, cucumber, watermelon and tea waste [11]. A modification or a pretreatment step of the cellulose-containing agricultural waste is advisable in sorption applications, to enrich the functional groups and increase the surface area and porosity [12]. The pretreatment step can be performed by several ways such as alkalization [13], acidification [14], esterification [15], etherification [16], carbonization [17], magnetization [18], and grafting [19]. The performance is expected to be better by using composites of the biomass with nanomaterials.

Perovskites nanomaterials are mixed metal oxides of the general formula ABO_3_, where A is a lanthanide and B is a transition metal. Due to the highly stabilized B metal in the perovskite matrix, perovskites possess many interesting structural, electrical, magnetic, optical properties [20–22]. Perovskites are highly recommended candidates in several important application, such as catalysis [23], sensors [24], capacitors [25], energy-storage [26], optical [27], and electrical devices [28], etc. The use of perovskites as sorbents for the removal of organic and inorganic pollutants is recently, but not too many, reported [29–31]. In our previous work, we used lanthanum-iron based-perovskite, prepared by the cellulose-modified method, for the removal of organic congo red dye [32], and inorganic toxic heavy metal ions; Pb(II), Cd(II), and Cu(II) ions [33]. The cellulose-modified method is performed by adding a raw or modified agricultural waste containing cellulose during the early stage of the perovskite synthesis. The product is perovskite-cellulose/biochar composite, rather than a pure perovskite [32].

In this work, LaFeO_3_ perovskite is prepared by the cellulose-modified microwave-assisted citrate method using two types of pretreated biomasses; rice straw (RS) and banana peel (BP). The effect of changing the biomass type on structural and surface properties of the prepared materials; LaFeO_3_/cellulose-RS and as LaFeO_3_/cellulose-BP are examined. Both samples are applied as sorbents for the removal of Pb(II) ion, the effect of the pretreatment step as well as changing the biomass type on the adsorption efficiency is investigated. The possibility of the sorbent regeneration and reuse, as well as, the performance in the real sample is studied.

## Experimental

### Chemicals

Ferric nitrate nonahydrate (98%), lanthanum (III) nitrate hexahydrate (99%), citric acid (98%), nitric acid (69%), acetic acid glacial ≥ 99%, ammonium hydroxide solution (30–33%), sodium hydroxide ≥ 98%, sodium chlorite (80%), lead nitrate (99%), cadmium chloride hydrate (98%), and copper sulfate pentahydrate (98%) are bought from Sigma-Aldrich. Bromocresol green is bought from Qualikems.

### Pretreatment of biomass

Cellulose is isolated by pretreatment of two different biomasses, rice straw (RS) and banana peel (BP) as reported [34]. Briefly, biomass is washed, dried, and grounded into a powder. The biomass powder is heated with 12 wt% of NaOH at 120 °C for 1 h to purify cellulose from lignin and hemicellulose. The solution is centrifuged and washed with distilled water. The residual is dried and added to acidified 5 wt% sodium chlorite at 75 °C for 90 min. The residual, α-cellulose, is washed with distilled water and dried at 40 °C overnight.

### ***Synthesis of LaFeO***_***3***_*** by the cellulose-modified citrate microwave-assisted method***

Ferric nitrate nonahydrate and lanthanum (III) nitrate hexahydrate are weighed in an equal molar ratio and dissolved in distilled water. Cellulose from either RS or BP is added to the mixed metals ions solution, and shaken for 24 h. The pH value is adjusted at 8 by 1 mmol L^−1^ nitric acid and 1mmol/L^−1^ ammonia solution, then citric acid is added in a molar amount equals to the total molar amount of metal ions. The mixed complex/cellulose suspension is heated till evaporation then placed in the microwave oven (720 W) for 30 min (20-s on and 10-s off). After dryness, the residual is ignited and finally calcinated at 450 °C for 3 h to obtain LaFeO_3_/cellulose-RS and LaFeO_3_/cellulose-BP materials [32, 33].

### Adsorption experiment

50 mg of LaFeO_3_/cellulose material is added to 25 mL of Pb(II) ion solution with an adjusted pH value at 7. The solution is shaken for 24 h, then it is filtered through 0.45 μm nylon syringe filter. The concentration of unadsorbed Pb(II) ions is determined by the atomic absorption spectroscopy (NovAA 350).

The removal % and the amount of adsorbed Pb(II) ions, *q*_*e*_, are determined from Eqs. (1) and (2), respectively [35]:

1 $$ Removal\, \% = \frac{{C_{o} - C_{e} }}{{C_{o} }} \times 100 $$

2 $$ q_{e} = \frac{{C_{{ads \times V_{L} }} }}{m} $$

where, *C*_*o*_, *C*_*e*_, and *C*_*ads*_ are initial, remaining, and adsorbed concentrations of Pb(II) ions (mg/g), respectively. *V*_*L*_ is the volume of the solution (L), and *m* is the mass of the adsorbent (g).

### Characterization techniques

Raman spectroscopy, Horiba labRAM HR evolution visible single spectrometer is used for structural identification. The particle size distribution is determined by the zeta seizer instrument (NanoSight NS500, Malvern Panalytical) by dynamic light scattering (DLS) method. Scanning electron microscope (SEM), (JEOL JXA-840A) is used for the morphological characterization. Brunauer–Emmett–Teller (BET) surface area is calculated using N_2_ gas as an adsorbate at 77 K, and done by Nova Touch, Quanta Chrome.

## Results and discussion

### Structural and surface characterizations

#### Raman spectroscopy

**Fig. 1:**
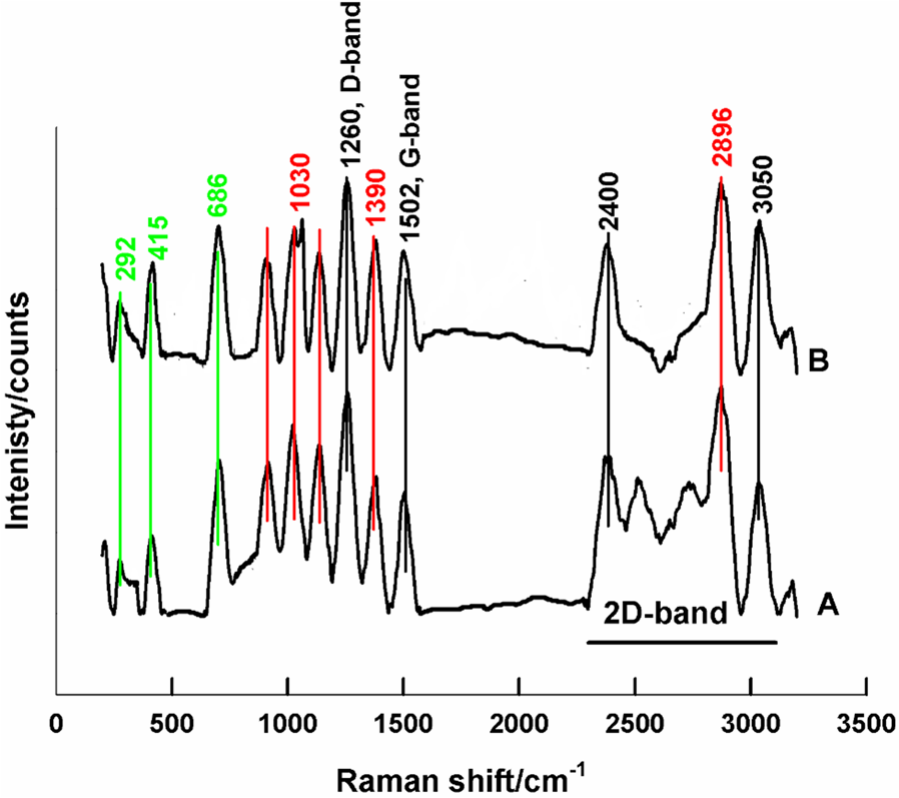
Raman spectra of LaFeO_3_/cellulose-RS (**A**), and LaFeO_3_/cellulose-BP (**B**)

Raman spectroscopy is used for the structure identification of prepared samples, Fig. 1 shows Raman spectra of LaFeO_3_/cellulose-RS (A), and LaFeO_3_/cellulose-BP (B). The formation of the octahedral LaFeO_3_ perovskite phase is ascertained by the appearance of bands at 292, 415, and 686 cm^−1^, which correspond to oxygen octahedral tilt, bending, and stretching vibration, respectively [36]. Characteristic cellulose bands are located around 1030 cm^−1^, and are assigned for symmetric and asymmetric stretching vibration of β-(1,4)-glycosidic linkage. Bands located at 1390 and 2896 cm^−1^ are assigned for CH_2_ bending and stretching modes, respectively [37]. The intensities of these band can be correlated to the crystallinity arrangement as well as the cellulose chain length. It can be noticed that cellulose sample prepared by using banana peel shows a longer chain length and a comparable crystallinity to that prepared by using straw rice. The appearance of the biochar phase, due to the thermal treatment of cellulose, is also detected by bands located at 1260 cm^−1^, 1502 cm^−1^, and band located between 2400 to 3050 cm^−1^. These bands correspond to D-, G-, and 2D-bands, respectively [38].

#### Scanning electron microscope (SEM) and particle size distribution

**Fig. 2:**
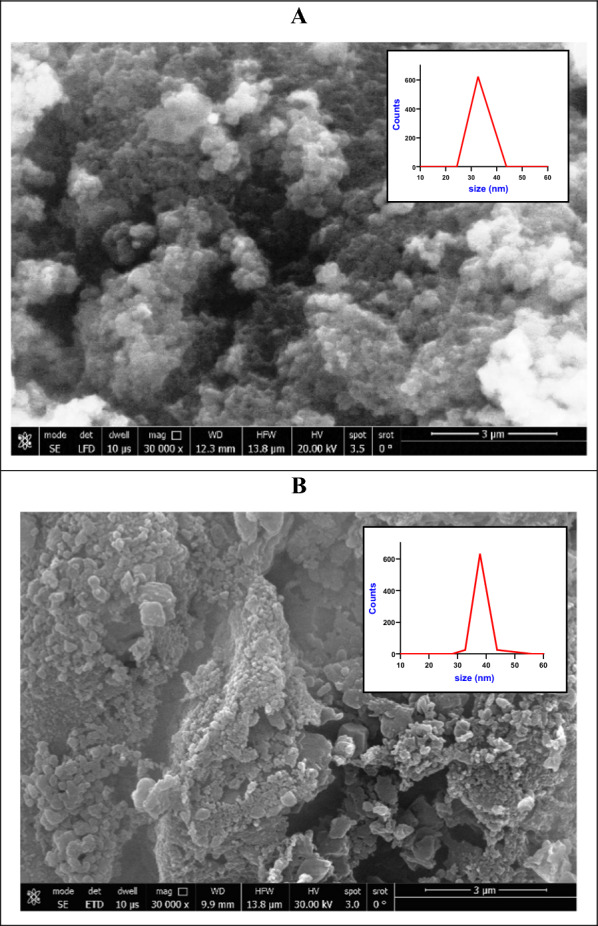
SEM images of LaFeO_3_/cellulose-RS (**A**), and LaFeO_3_/cellulose-BP (**B**). The insets are the corresponding particle size distribution, estimated by DLS method

Figure 2 shows SEM images of LaFeO_3_/cellulose-RS (A), and LaFeO_3_/cellulose-BP (B). The insets represent the corresponding particle size distribution, estimated by DLS method. LaFeO_3_/cellulose-RS has a cauliflower-like morphology, Fig. 2A. While LaFeO_3_/cellulose-BP has a different morphology, in which two phases can be assigned; mesoporous phase of cellulose and biochar, and nanoparticle of octahedral LaFeO_3_ perovskite, Fig. 2B. The average particle size values are 32 and 38 nm for LaFeO_3_/cellulose-RS and LaFeO_3_/cellulose-BP, respectively, insets of Fig. 2.

#### BET surface area measurements

**Fig. 3:**
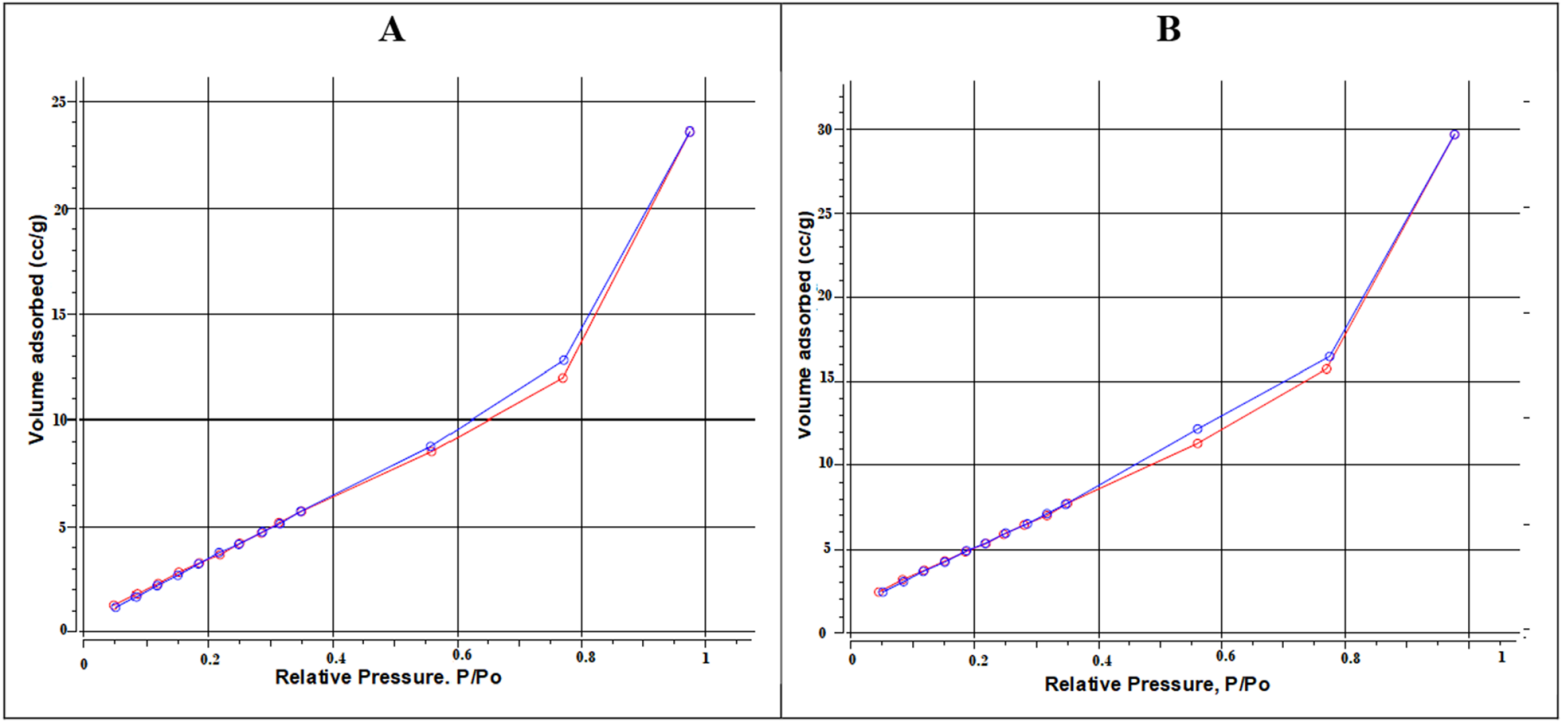
BET hysteresis loops for nitrogen adsorption/desorption on LaFeO_3_/cellulose-RS (**A**), and LaFeO_3_/cellulose-BP (**B**)

Nitrogen adsorption/desorption hysteresis loops for the prepared samples are shown in Fig. 3. Both loops are H3 types, which means that both samples; LaFeO_3_/cellulose-RS, and LaFeO_3_/cellulose-BP have pores of a slit-like shape [39, 40]. The pore size and pore volume as well as the surface area are calculated by the BET method and listed in Table 1. It can be shown LaFeO_3_/cellulose-RS sample has smaller pore size and pore volume than LaFeO_3_/cellulose-BP sample, which indicated the decreased sample porosity by using cellulose prepared from RS. The calculated BET surface areas are 20.99 and 24.58 m^2^/g for LaFeO_3_/cellulose-RS and LaFeO_3_/cellulose-BP, respectively. According to the particle size distribution profiles of the two samples, insets of Fig. 2, LaFeO_3_/cellulose-RS sample has a smaller particle size of 32 nm as compared to that of LaFeO_3_/cellulose-BP sample, 38 nm. It can be concluded that the former has a lower porosity as indicated by its smaller particle size and decreased surface area with respect to the banana peel-based perovskite sample.

**Table 1:** Average pore size and volume as well as measured surface area calculated by BET method for the prepared samples

Sample	BET surface m^2^/g	Total pore volume (cc/g)	Average pore size (nm)
LaFeO_3_/cellulose-RS	20.99	0.0366	3.49
LaFeO_3_/cellulose-BP	24.58	0.0460	3.74

### ***Application of cellulose-modified LaFeO***_***3***_*** samples as sorbents for the removal of Pb(II) ions from aqueous solutions***

#### Effect of the initial Pb(II) ion concentration

**Fig. 4:**
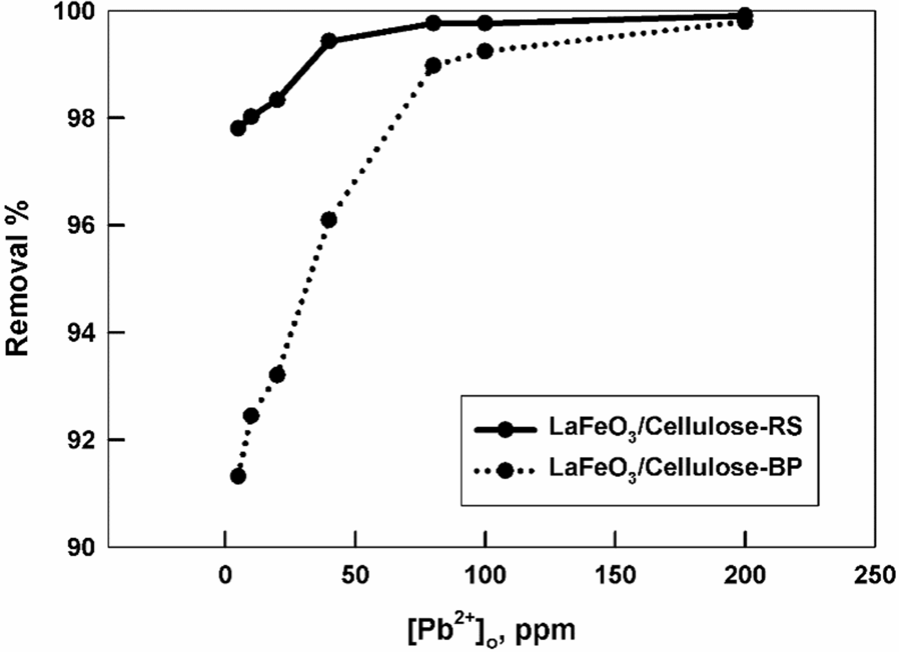
The variation of the removal % of LaFeO_3_/cellulose-RS and LaFeO_3_/cellulose-BP for Pb(II) ions at pH = 7 for a shaking time = 2 h at room temperature

The adsorption experiments of Pb(II) ion on each of the prepared samples, LaFeO_3_/cellulose-RS, and LaFeO_3_/cellulose-BP, are conducted at different initial Pb(II) ion concentration, ranging from 5 to 200 ppm, at pH = 7 and room temperature. Figure 4 shows the dependence of the removal % of Pb(II) ions by perovskite/cellulose samples on the initial metal ion concentration. The removal % is high, > 90% and increases with increasing the initial metal ion concentration, which indicates the availability of active adsorption sites [41]. LaFeO_3_/cellulose-RS offers a better removal performance for Pb(II) ions at low initial metal ion concentration with respect to LaFeO_3_/cellulose-BP, while at high concentration ≥ 100 ppm, both samples exhibit a comparable sorption ability.

#### Adsorption isotherms

**Fig. 5:**
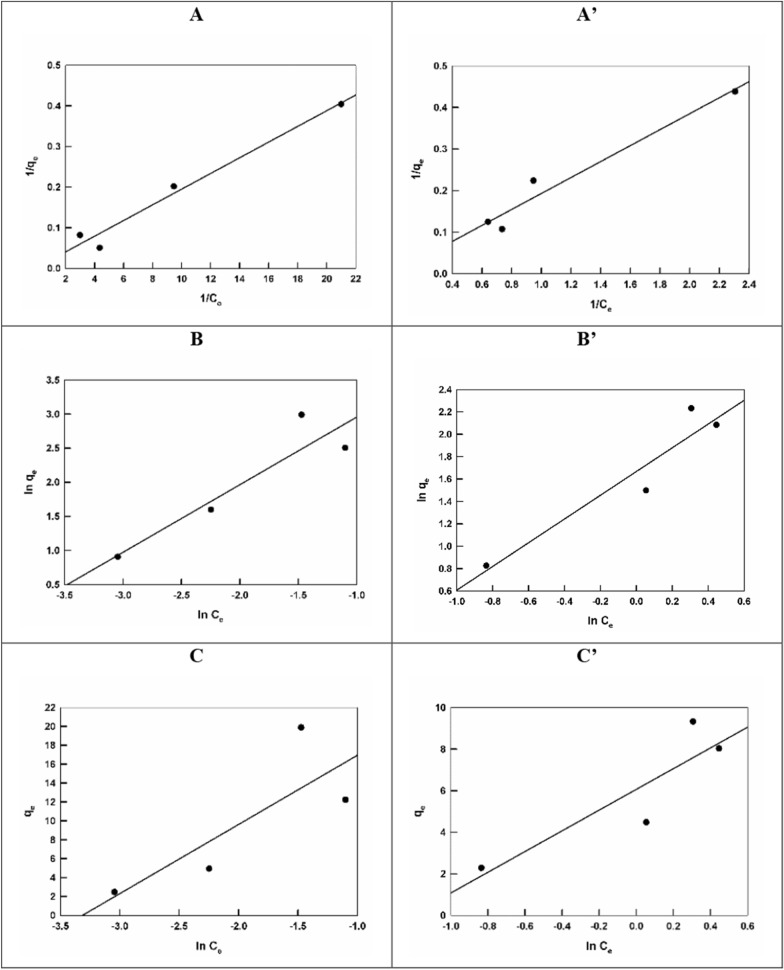
Langmuir (**A**, **A**’), Freundlich (**B**, **B**’), and Temkin (**C**, **C**’) isotherms for the adsorption of Pb(II) ions of LaFeO_3_/cellulose-RS and LaFeO_3_/cellulose-BP, respectively

For a better elucidation of the adsorption mechanism, the adsorption data of Pb(II) ions, on perovskites samples modified with cellulose from different biomasses; RS and BP, are fitted to different isotherms; Langmuir, Freundlich, and Temkin isotherms. While Langmuir and Freundlich isotherms suppose that there is no lateral interaction, Temkin isotherm assumes the existence of adsorbate-sorbent interaction. Linear forms of Langmuir, Freundlich, and Temkin isotherms can be expressed by the following equations, respectively [42]:

3 $$ \frac{1}{{q_{e} }} = \frac{1}{{q_{m} }} + \frac{1}{{q_{m}\, K_{L} C_{e} }} $$

4 $$ ln\; q_{e} = ln\; K_{F} + \frac{1}{n}\; ln\; C_{e} $$

5 $$ q_{e} = \frac{RT}{b}lnK_{T} + \frac{RT}{b}\ln C_{e} $$

where *q*_*m*_ is the maximum adsorption capacity (mg/g), *K*_*L*_, (. *K*_*F*_ and *n*), and (*K*_*T*_ and *b*) are Langmuir, Freundlich, and Temkin constants, respectively. Figure 5 shows the different adsorption isotherms for the adsorption of Pb(II) ions on perovskites samples modified with cellulose prepared from RS and BP. Table 2 summarizes the calculated parameters from the different isotherms for the adsorption of Pb(II) ions on the two proposed sorbents.

**Table 2:** Adsorption parameters calculated by fitting Langmuir, Freundlich, and Temkin isotherms to the adsorption data of Pb(II) ions on LaFeO_3_/cellulose-RS and LaFeO_3_/cellulose-BP

Sorbent	Langmuir isotherm			Freundlich isotherm			Temkin isotherm		
	*q*_*m*_ (mg/g)	*K*_*L*_ (L/mg)	*r*^*2*^	*n*	*K*_*F*_ (mg^1−(1/n)^ L^1/n^/g)	*r*^*2*^	*b* (kJ/mol)	*K*_*T*_ (L/mg)	*r*^*2*^
LaFeO_3_/cellulose-RS	523.56	0.096	0.9730	1.01	51.94	0.8487	338.82	27.94	0.6469
LaFeO_3_/cellulose-BP	729.93	0.007	0.9580	0.94	5.31	0.9072	496.67	3.39	0.7871

Based on the correlation coefficient, *r*^*2*^, values, the adsorption data fits better Langmuir isotherm for both, LaFeO_3_/cellulose-RS and LaFeO_3_/cellulose-BP. This means that the adsorption of Pb(II) ions on perovskite modified-cellulose samples is a monolayer adsorption in which adsorbed Pb(II) ions form coordination bonds with the functional groups at the sorbent surface [43]. The same mechanism is verified, irrespective to the biomass type used as a source for the cellulose precursor. However, the calculated maximum adsorption capacities, *q*_*m*_, are 523.6 and 729.9 mg/g for LaFeO_3_/cellulose-RS and LaFeO_3_/cellulose-BP, respectively. BP is more recommended as a biomass for cellulose preparation in this work. This is due to longer chains, and increased porosity and surface area offered by LaFeO_3_/cellulose-BP as compared to LaFeO_3_/cellulose-RS. It was reported that the adsorption of Pb(II) ions on biochar prepared from RS is based on chemical complexation mechanism with *q*_*m*_ value of 176.1 mg/g [44], while for biochar prepared from BP, the *q*_*m*_ value is 247.1 mg/g [45]. This finding agrees well with the sorption performance reported in this work. In a previous work, we used BP during the perovskite synthesis without the pretreatment step [32]. The calculated *q*_*m*_ value for the adsorption of Pb(II) ions, is 606.1 mg/g, which is smaller than that reported in this work. Therefore, it is recommended to perform the pretreatment step of the biomass before the perovskite synthesis.

#### Regeneration and reuse

**Fig. 6:**
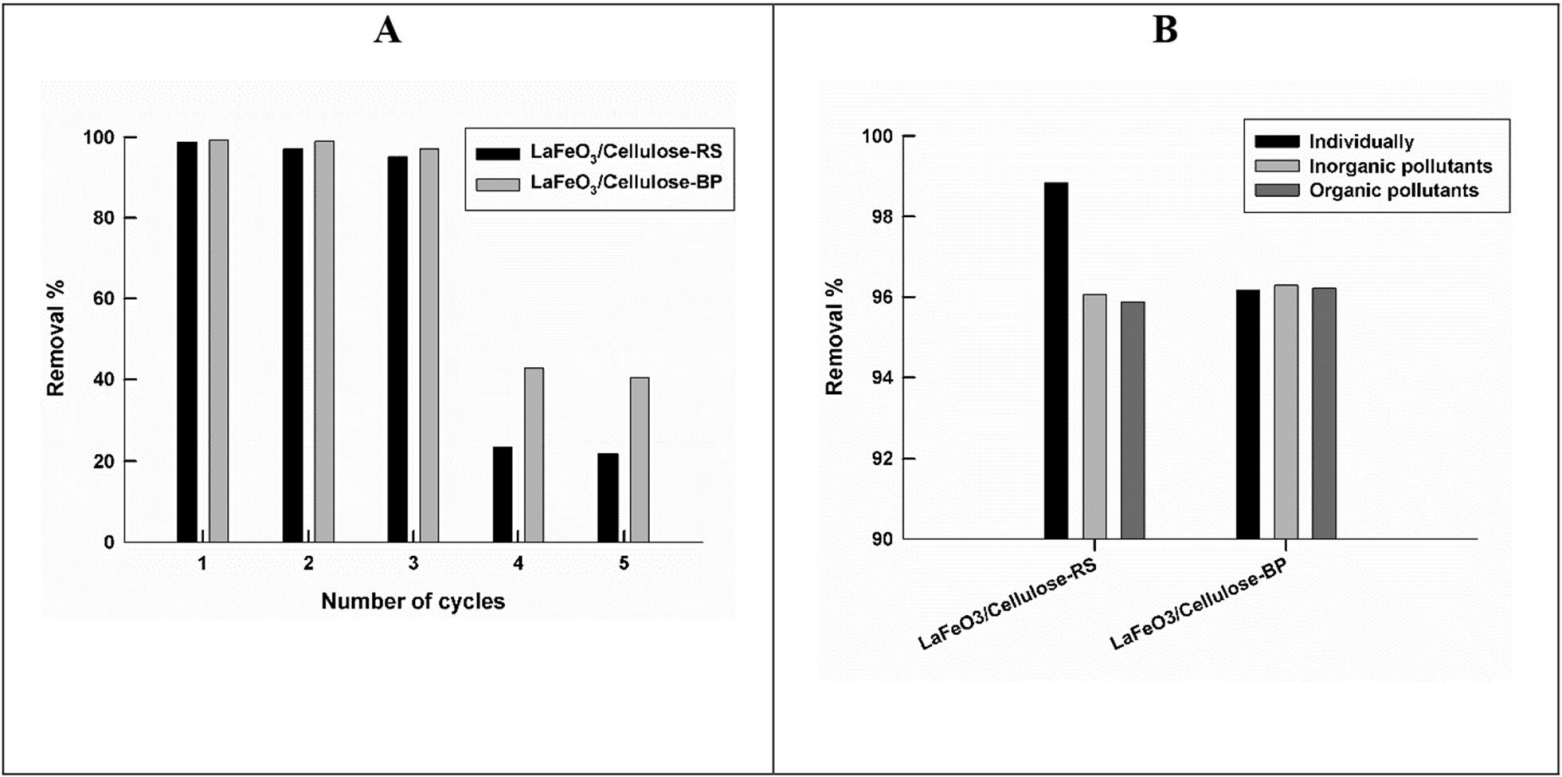
The variation of the removal % of Pb(II) ions by LaFeO_3_/cellulose-RS and LaFeO_3_/cellulose-BP, with successive regeneration and reuse cycles (**A**), In the absence and presence of inorganic or organic pollutants (**B**)

The possibility of the sorbent regeneration and reuse for the remove of Pb(II) ions are checked for the two prepared samples LaFeO_3_/cellulose-RS and LaFeO_3_/cellulose-BP. Sorbents are regenerated by being shaken in 1% HNO_3_ solution for 24 h, then samples are washed with distilled water and dried at 40 °C for 24 h, before reuse [32]. Figure 6A shows the variation of the removal % of Pb(II) ions by LaFeO_3_/cellulose-RS and LaFeO_3_/cellulose-BP, with repeated regeneration and reuse cycles.

Both sorbents can be regenerated and reused successively for 3 cycles, the decrease of the removal % by performing three successive usage cycles are 3.6 and 2.3% for LaFeO_3_/cellulose-RS and LaFeO_3_/cellulose-BP, respectively. Starting from the 4th cycle, the sorption efficiency is decreased to about 20%, of the value calculated for the fresh LaFeO_3_/cellulose-RS sample, and decreased to about 40% of the value calculated for the fresh LaFeO_3_/cellulose-BP sample. This reflects that the latter is a better sorbent. The decrease in the adsorption ability by successive cycles of adsorption and desorption can be due to the accumulation of impurities or contaminants on the surface that block the active sites or pores of the adsorbent or the degradation or deterioration of the adsorbent structure due to thermal, chemical, or mechanical stress.

#### Interferences study

The sorption performance of LaFeO_3_/cellulose-RS and LaFeO_3_/cellulose-BP for Pb(II) ion is checked firstly, in the presence of other heavy metal ions; 30 ppm Cu(II) and 30 ppm Cd(II) ions, as inorganic pollutants. Secondly, in the presence of 30 ppm bromocresol green dye, as an organic pollutant. The removal % of the two proposed sorbents, for Pb(II) ion, is compared for the three cases; when Pb(II) ions present individually, in the presence of inorganic pollutants, and in the presence of organic pollutants, as shown in Fig. 6B. The removal % of LaFeO_3_/cellulose-RS for Pb(II) ions is slightly decreased by the presence of inorganic and organic pollutants (decreased by about 3% as compared to the individual case). While, The removal % of LaFeO_3_/cellulose-BP for Pb(II) ions is not affected by the presence of inorganic and organic pollutants. Therefore, both sorbents can be used effectively for the removal of Pb(II) ions without being affected by the matrix of the real sample, and highly recommended for the field applications.

## Conclusion

- LaFeO_3_ perovskite can be successively prepared by the cellulose-modified microwave-assisted citrate method. The product is a composite of LaFeO_3_, cellulose, and biochar, as confirmed by Raman spectroscopy. The appearance of oxygen octahedral bands, vibration of β-(1,4)-glycosidic linkage, and D-, G-, and 2D-bands, respectively.
- Changing the biomass used as a cellulose source, either RS or BP, affect the structure, morphology, and surface area of the prepared perovskite. LaFeO_3_/cellulose-RS (20.99 m^2^/g) has a cauliflower-like morphology, while LaFeO_3_/cellulose-BP (24.58 m^2^/g) has two phases; mesoporous cellulose and biochar, and perovskite nanoparticles.
- The prepared LaFeO_3_/cellulose-RS and LaFeO_3_/cellulose-BP are applied as sorbents for the removal of Pb(II) ions from aqueous solution. Adsorption follows Langmuir isotherm, and calculated *q*_*m*_ values are 523.6 and 729.9 mg/g for LaFeO_3_/cellulose-RS and LaFeO_3_/cellulose-BP, respectively.
- Proposed sorbents can be effectively regenerated and reused for successive three cycles, and can perform efficiently in the real sample matrix.
- Both sorbents exhibit an excellent sorption performance with a preferred direction to use of BP as a cellulose source during the sorbent synthesis due to the better adsorption efficiency and higher selectivity.

## Data Availability

The data and materials of this research are available by requesting from the corresponding author.
